# 문제중심학습 통합 시뮬레이션교육이 간호대학생의 간호지식, 비판적 사고성향, 문제 해결 능력 및 수업 몰입도에 미치는 효과

**DOI:** 10.4069/kjwhn.2020.03.15.1

**Published:** 2020-03-25

**Authors:** Young A Song

**Affiliations:** Department of Nursing, Ansan University, Ansan, Korea; 안산대학교 간호학과

**Keywords:** Problem-based learning, Simulation, Critical thinking, Problem-solving, 문제중심학습, 시뮬레이션, 비판적 사고, 문제 해결

## Abstract

**Purpose:**

This study was conducted to investigate the effects of problem-based learning–integrative simulation practice (PBL-ISP) on nursing knowledge, critical thinking, problem-solving ability, and immersion among nursing students.

**Methods:**

The study used a nonequivalent control group post-test design. A PBL-ISP educational program was provided to the experimental group, and hands-on practice using a high-fidelity simulator (HFS) was administered to the control group. There were 42 subjects in the experimental group and 40 in the control group. Data were collected during the fall semester of 2019. Using SPSS for Windows version 23.0, data were analyzed in terms of frequency, percentage, mean, standard deviation, the chi-square test, t-test, and Pearson correlation coefficients.

**Results:**

Significant differences between the experimental and control groups were found in nursing knowledge (t=3.67, *p*<.001), critical thinking (t=3.40, *p*=.001), problem-solving ability (t=3.52, *p*=.001) and immersion (t=4.44, *p*<.001).

**Conclusion:**

PBL-ISP was more effective in improving nursing knowledge, critical thinking, problem-solving ability, and immersion than was hands-on practice using an HFS.

## Introduction

### 연구 필요성

문제중심학습은 학생들에게 학습 동기를 유도하고 임상적 맥락 안에서 지식을 구조화하며 자기 성취감을 촉진하고 문제 해결 등의 인식력을 높인다. 특히 학생들로 하여금 비판적, 성찰적 사고 습관을 실천하게 하고 학습의 책임감과 자율성을 높이는 교수 학습전략이다[1]. Cannon과 Schell [2]에 의하면, 간호사들에게 필요한 것은 여러 다양한 간호 상황과 과정 중에 필요한 과학적인 문제 해결 및 대처 능력이라고 하였다. 특히, 학생 본인이 지속적으로 간호 전문성을 갖추기 위해서는 학습에 대한 자기주도력(self-directed learning)이 필요하다고 하였다. 즉 자신의 학습의 진행 방향, 내용, 결과 등에 대하여 직접적이고 주도적인 역할을 할 수 있어야 한다[3].

간호교육 과정에서 실습교육은 학생들이 관련 지식의 통합적 사고를 통해 환자의 간호 문제를 파악하고 근거 있는 간호 진단을 내려 정확하게 간호 수행을 할 수 있도록 이루어져야 한다[4]. 특히, 임상 현장에서는 다양한 상황에서 비판적 사고를 적용하여 문제를 해결하도록 요구한다. 그러나 임상의 특성과 환경을 살펴보면 주로 관찰 위주의 실습이 이루어져 간호 역량을 키우기가 점점 어려워지고 있다[5]. 이러한 상황을 극복하기 위해 시뮬레이션을 활용한 다양한 실습교육이 개발되고 있다. 선행연구[6-8]에 의하면, 고충실도 환자 모형 시뮬레이터(high-fidelity patient simulator)를 활용한 실습교육, 표준화 환자를 활용한 실습교육 등에 대한 국내 연구들에서 간호 수행 능력, 의사소통 능력, 임상 판단 및 관리 능력, 지식 습득, 수업 만족도, 비판적 사고, 문제 해결 능력 등이 향상되었다고 보고하였다. 국외 연구에서도 비판적 사고, 문제 해결 과정, 임상 수행 능력, 의사결정 능력 등의 향상 결과를 보고하였다[9,10]. 또한 문제중심학습을 융합한 시뮬레이션 실습교육의 효과를 연구한 선행논문에서도 유사한 결과를 보였다[11-15].

오늘날 간호학생들의 산부인과 임상 실습이 저출산과 모아의 인권 존중 등을 이유로 매우 어려워진 실정이다[16]. 즉, 간호대학들의 임상 실습 의료기관 부족 문제와 더불어 환자 권리 강화로 인해 실습 기회가 부족하고 더 나아가 최근에는 관찰 위주의 실습 기회조차 감소하고 있는 실정이다[17]. 이와 같은 문제를 해결하기 위한 방안으로 다양한 시뮬레이션 실습교육을 적용하고 있고, 그 효과를 파악하는 연구의 중요성이 대두되고 있다[11]. 또한 시뮬레이션 실습교육의 한 유형으로 분만 시뮬레이터를 활용해서 분만실에서 경험할 수 있는 다양한 상황에서 학생들의 실습교육을 실시함으로써 교육의 효과를 거두었다고 보고한 연구도 있다[18]. 이러한 이유로 오늘날 많은 대학들이 시뮬레이션 실습실과 음향 및 장비 체계를 구축하고 있으며, 시뮬레이션 실습이 교과목으로 개설되고 있다[19]. 이러한 시뮬레이션 기반 학습은 안전하게 설정된 가상 환경에서 환자에게 제공된 간호 및 의사결정의 결과를 관찰할 수 있는 기회를 제공하고, 팀 워크와 의사소통 능력 등을 함양할 수 있는 전략을 제공할 수 있다[19]. 그러므로 시뮬레이션 실습을 통해 얻어진 임상 수행 기술과 의사결정 능력으로 졸업 후 간호사들이 임상에 쉽게 적응하고 습득한 간호지식을 적절하게 적용하도록 해주는 것이다. 이러한 시뮬레이션 학습의 특성으로 인해 간호교육에서 지식과 기술 등의 통합 교육으로서 시뮬레이션 실습교육이 학습자들의 간호 역량을 함양할 수 있는 교수 전략으로 활용된다[4]. Song [11]은 간호학생의 간호 수행 능력을 향상시키는 요인이 우선 문제중심학습을 적용하여 문제 해결 과정을 익힌 후 임상 현장과 유사한 상황에서의 시뮬레이션 반복 실습교육을 실시하는 것이라고 보고한 바, 문제중심학습과 시뮬레이션 실습의 이점을 통합하여 적용한다면 그 효과를 극대화할 수 있을 것이다[12,13].

최근 시뮬레이션 실습을 일부 임상 실습교육 시간으로 허용하여 대학 인증평가에도 반영하고 있다. 따라서 최대한 임상 상황과 유사하게 재현하기 위해 고충실도 시뮬레이터(high-fidelity simulator)를 활용한 실습이 널리 이루어지고 있다[20]. 그러나 시뮬레이션 실습을 통해 학생들의 역량을 극대화하기에는 몇몇 문제점이 나타나고 있다[21]. 즉 임상과 최대한 유사한 시나리오 개발, 교수자들의 역량 및 인력 풀 구축, 학생들의 비판적 사고 능력과 문제 해결 능력, 의사소통 능력 및 의사결정 능력 등을 강화할 수 있는 시뮬레이션 실습교육 프로그램의 개발이 중요하다고 생각한다. 이에 본 연구에서는 학생들이 수업에 몰입하여 최대한 학생들이 가지고 있는 능력을 발휘할 수 있는 실습 환경과 실습의 인력 풀 및 실제 상황에 최대한 근접한 현실성 있는 문제중심학습 통합 시뮬레이션 실습교육(problem-based learning–integrative simulation practice, PBL-ISP) 모듈을 적용하고 그 효과를 알아보고자 한다.

궁극적으로 본 연구는 임상에서 요구하는 핵심능력을 향상시키고, 간호사가 다방면으로 갖추어야 할 역량을 기를 수 있도록 통합 시뮬레이션 실습교육 프로그램을 개발하고 적용하고자 한다. 또한 어떻게 수업을 진행하는 것이 학생들에게 실제적 도움을 주며 더 긍정적인 효과를 기대할 수 있는지 방안을 제시할 필요가 있다고 생각하므로, 본 연구는 임상 실습의 효과만큼 기대할 수 있도록 기존의 고충실도 시뮬레이터를 활용한 실습교육과 비교함으로써 PBL-ISP 프로그램의 공유와 확대를 위한 기초 자료를 제시하고자 한다.

### 연구 목적 및 가설

본 연구의 목적은 PBL-ISP 교육을 받은 실험군과 고충실도 시뮬레이터를 활용한 실습교육을 받은 대조군 간의 간호지식, 비판적 사고성향, 문제 해결 능력 및 수업 몰입도의 차이를 파악하는 것이다.

가설 1. 실험군은 대조군보다 간호지식 점수가 높을 것이다.

가설 2. 실험군은 대조군보다 비판적 사고성향 점수가 높을 것이다.

가설 3. 실험군은 대조군보다 문제 해결 능력 점수가 높을 것이다.

가설 4. 실험군은 대조군보다 수업 몰입도 점수가 높을 것이다.

### 용어 정의

#### PBL-ISP 교육

문제중심학습은 구성주의에 바탕을 둔 교수-학습이론을 기반으로 하는 학습모형이며, PBL은 학습자들이 문제를 바탕으로 해결책을 마련하기 위해 필요한 지식을 자율적으로 수집하고 활용하는 과정으로 문제 해결을 위해 타인과 협력하여 해결 과정을 이끌어가는 자기주도적 학습이다[11]. 시뮬레이션 실습교육은 임상 현장과 유사한 상황에서 문제를 직접 해결하면서 학습이 일어나도록 하는 교수 학습법이다[18]. 본 연구에서 PBL-ISP는 문제중심학습 모듈과 시뮬레이션 모듈을 통합하여 개발한 통합 시뮬레이션 실습교육 프로그램을 의미한다. PBL-ISP 교육에서 활용하는 환자 시뮬레이터(human patient simulator)는 계획되고 구조화된 시나리오로 환자의 임상 상황을 구현할 수 있는 고충실도 시뮬레이터 또는 표준화 환자(standardized patient)를 말하며, 직접적인 상호작용 및 임상 개입을 훈련할 수 있는 시뮬레이션 실습교육을 의미한다.

#### 고충실도 시뮬레이터를 활용한 실습교육

고충실도 시뮬레이터는 병리학적 환자 상황을 모방할 수 있는 현실적인 마네킹으로, 임상 결과를 입증하고 의사소통하며 약물 투여 및 절차와 같은 간호중재에 대응할 수 있는 시뮬레이터이다[20]. 본 연구에서 고충실도 시뮬레이터는 다양한 측면에서 사람과 유사한 반응을 나타낼 수 있는 것으로 시나리오를 기반으로 프로그래밍한 알고리즘을 SimMan, SimBaby와 SimMom 시뮬레이터(Laerdal Medical, Stavanger, Norway)를 활용하여 임상 상황을 재현하는 시뮬레이션 실습교육을 의미한다.

## Methods

Ethics statement: This study was approved by the Institutional Review Board of Ansan University (AN01-201812-HR-011-01). Informed consent was obtained from the subjects.

### 연구 설계

**Figure. 1.**
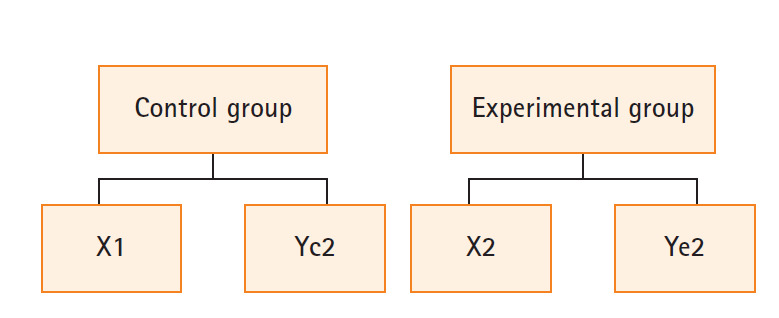
Research design. X1=high-fidelity simulation method; X2=problem-based learning–integrative simulation practice method; Yc2, Ye2=post-test (nursing knowledge, critical thinking, problem-solving ability, and immersion).

본 연구는 PBL-ISP 교육을 받은 실험군과 고충실도 시뮬레이터를 활용한 실습교육을 받은 대조군 간의 간호지식 점수, 비판적 사고성향, 문제 해결 능력 및 수업 몰입도의 차이를 비교하기 위해 비동등성 대조군 사후시차 설계를 적용한 유사실험연구(nonequivalent control group non-synchronized post-test design)이다. 학기별 학생을 두 그룹으로 구분하기 때문에 실험의 확산을 예방하기 위하여 우선 대조군에게 처치 후 사후조사를 실시하고, 이어서 실험군에게 처치와 사후조사를 실시하였다(Figure 1).

### 연구 대상

본 연구 대상자는 미국 캘리포니아주 M 대학 간호학과 1학기에서 4학기에 재학 중인 학생으로, 간호학과 입학 전 1년 동안 해부학, 생리학, 미생물학, 병리학의 기초 교과목을 이수한 후 진급 시험을 통해 간호학과에 입학한 학생 82명을 대상으로 하였다. 실험군과 대조군 구분은 2019년 가을 학기에 실시하는 시뮬레이션 실습 시 1학기에서 4학기 학생들의 실습계획표에서 먼저 실습에 배정된 학생을 대조군으로 하여 40명을 선정하였고, 뒤에 배정된 학생 42명을 실험군으로 선정하였으며, 연구 참여에 동의한 학생을 편의표출 하였다.

표본수 산정을 위해 G*power 3.1.9.6 프리웨어를 이용하여 independent t-test를 위한 대상자를 유의수준 α는 .05, 효과 크기 0.7, 검정력을 0.8로 했을 때, 연구에 필요한 표본수는 각 그룹에 26명이었다. 본 연구에서는 실습계획표에 이미 배정된 학생 전수로 중도 탈락자 없이 실험군 42명, 대조군 40명의 총 82명을 대상으로 하였다.

### 연구 도구

#### 간호지식

총 4개 모듈의 간호지식을 측정하기 위해, 자료를 수집한 대학의 교과목 교수 4명이 개발한 기존 도구의 사용을 허락받았다. 이 도구들은 A형의 문제와 문제 해결을 요구하는 문제로 총 20개의 문항으로 구성되어 있다. 각 문항에 대해 정답은 1점, 오답은 0점으로 처리하였고 점수의 범위는 최고 20점–최저 0점으로 점수가 높을수록 간호지식 점수가 높은 것을 의미하며, 시뮬레이션 실습교육 후 자가 기입 방법으로 작성하였다. 본 연구에서 도구의 신뢰도 Cronbach’s α는 .61이었다.

#### 비판적 사고성향

본 연구에서는 Yoon [22]이 개발한 비판적 사고성향 측정도구를 사용하였다. 비판적 사고성향 도구는 지적/열정/호기심 5문항, 신중성 4문항, 자신감 4문항, 체계성 3문항, 지적 공정성 4문항, 건전한 회의성 4문항, 객관성 3문항으로 이루어진 총 27문항의 5점 Likert 척도로 ‘전혀 그렇지 않다’ 1점에서부터 ‘매우 그렇다’ 5점으로 배점하였고, 총점은 27점에서 135점으로 점수가 높을수록 비판적 성향이 강한 것을 의미한다. Yoon [22]의 연구에서 Cronbach’s α는 .89였고, 본 연구에서는 .87이었다.

#### 문제해결능력

문제해결능력은 한국교육개발원에서 Lee 등[23]이 개발한 문제해결능력 진단지를 사용하였다. 이 도구는 문제 명료화 5문항, 원인 분석 10문항, 대안 개발 10문항, 계획/실행 10문항, 수행평가 10문항의 총 45문항으로 구성되어 있다. 각 문항에 대하여 ‘전혀 아니다’ 1점에서 ‘매우 그렇다’ 5점의 Likert 척도로 측정하며, 점수가 높을수록 문제해결능력이 높음을 의미한다. Lee 등[23]의 연구에서 Cronbach’s α는 .94였으며, 본 연구에서도 .94였다.

#### 수업 몰입도

수업 몰입도(Immersion) 평가도구는 Shin 등[24]이 개발한 도구를 이용하였다. 이 도구는 즐거움 5문항, 원격 현존감 3문항, 주의 집중 4문항, 관여 4문항, 시간 왜곡 4문항 등 총 20문항의 5점 Likert 척도로, ‘전혀 아니다’ 1점에서 ‘매우 그렇다’ 5점으로 배점 처리하였다. Shin 등[24]의 연구에서 Cronbach’s α는 .76이었으며, 본 연구에서는 .90이었다.

### 연구 진행절차

#### PBL-ISP 교육 모듈 개발

PBL-ISP 교육 모듈은 총 4개의 시나리오와 관련 자료, 문제 해결 접근방법, 전자의무기록(electronic medical record, EMR) 프로그램을 설정하고 부서와 상황에 따라 situation, background, assessment, recommendation (SBAR)을 준비하였다. 학생들은 다양한 SBAR 양식 중 간호사가 간호사에게 보고하는 SBAR와 간호사가 의사에게 보고하는 SBAR를 사용하였다. 선수 학습 내용 및 간호지식 질문지와 간호학과 핵심 술기 중에서 시나리오별 해당 간호술기 및 영상 매체, 표준화 환자 및 시뮬레이터 세팅, 교수와 학생 준비물 등으로 구성하였다.

PBL-ISP 교육 모듈 개발을 위해 대학에서 기존에 진행해 온 시뮬레이션 교육과 활용한 자료(시나리오, EMR 정보 등)를 모두 수집한 뒤 2019년 5월 16일에 정리한 내용을 토대로 M 대학의 시뮬레이션 전임 교수 1인과 학기 담당 교수(adult nursing, maternity nursing, pediatric nursing, dementia nursing, nursing management), 시뮬레이션 실습과 임상 실습 외래 교수, 임상 간호사 1인 등 총 8명과 함께 논의 및 수정을 통해 모듈에 포함할 내용을 의논하여 학기별 모듈과 핵심 간호술기 및 활용 가능한 시뮬레이터 등 총 4개의 모듈을 구성하였다(Table 1).

각 모듈은 PBL 모듈 개요(module introduction)와 알고리즘, ISP 모듈 개요, 평가자 체크리스트, 디브리핑(debriefing)으로 구성한다. PBL 모듈 개요에는 모듈 주제, 학습목표, 선수 학습 및 질문지, 시나리오 및 관련 정보, 문제 해결 접근방법으로 구성한다. 알고리즘은 시나리오 흐름에 따라 내용을 구성하는 것으로, 간호사의 중재와 간호 대상자 및 의료진 등의 상황 및 이에 따른 행동과 말로 이루어진다. ISP 모듈 개요에는 실습목표, 핵심 간호수기, 시뮬레이션 평가 임상 실습 시나리오, 시뮬레이션 구현을 위한 표준화 환자나 고충실도 시뮬레이터 세팅, 시뮬레이션 실습 병동에 투입할 팀 간호학생과 간호사 역할 시트, EMR 세팅과 SBAR 양식으로 구성하며, 평가자는 학생 2, 3명당 1인으로 두고, 평가자는 교수자, 임상 간호사 등으로 정한다. 평가자 체크리스트는 시뮬레이션 시나리오를 바탕으로 한 간호술기와 태도를 교수자가 평가할 수 있도록 되어 있으며, 마지막으로 간소 디브리핑(simplicity debriefing)과 통합 디브리핑(integral debriefing)으로 구성한다.

총 4개의 모듈은 PBL-ISP 교육 프로그램 10단계에 의해 6시간 동안 진행한다. PBL-ISP 교육을 단계별로 살펴보면, 다음과 같다. 1단계, 시뮬레이션 실습에서 다루어지는 핵심술기에 대한 동영상과 학습목표 및 관련 정보를 온라인에 탑재하여 선수 자가학습을 한다. 2단계, 수업 시간에 모듈과 관련 자료, physical order, SBAR 등을 제공하고 모듈에 따라 대상자의 간호문제를 해결하도록 안내한다. 3단계, 문제중심학습의 학습 이슈(learning issues)에 관한 자기주도 학습을 통해 지식, 기술, 태도를 습득하도록 한다. 4단계, PBL에서 제시한 시나리오를 구현하기 위해 간호사의 역할(책임, 교육, 치료, 처치, 관찰자, 기록자, 보호자[무전으로 교수와 소통] 등)을 상황에 맞게 분담하고 간호를 수행할 수 있도록 한다. 5단계, 시뮬레이션 실행(running) 동안 평가자(임상 간호사, 시뮬레이션 전임교수, 교과목 교수 등)의 감시 아래 표준화 환자 또는 고충실도 시뮬레이터 간호를 실시한다. 6단계, 간호사정 내용과 간호과정 등을 EMR에 기록하고 SBAR를 활용하여 보고한다. 7단계, 간소 디브리핑을 표준화 환자 및 평가자와 함께 10–15분 정도 실시한다. 8단계, 표준화 환자를 제외한 전체 학생과 교수들, 임상 간호사 등과 함께 통합 디브리핑을 한다. 9단계, 평가자들은 평가 결과를 공유하고, pass와 fail에 대한 정확한 근거를 교수 공유 사이트에 올리며 학생 개인에게 피드백을 한다. 10단계, fail 학생은 Open Lab 일정에 재실습 시간을 올리면 시뮬레이션 교수자의 감독하에 훈련 및 재시험의 기회를 얻는다. 병원에서 요구하는 진술서에 평가자의 pass 사인을 받아 제출 후 임상 실습에 참여한다.

#### 시뮬레이션 실습교육 운영

PBL-ISP 교육 프로그램은 가을 학기 시뮬레이션 실습에서 학기별 시뮬레이션 실습은 같은 실습내용으로 실습하였고, 실습교육의 방법에서 대조군에게 고충실도 시뮬레이터를 활용한 기존 실습교육을 실시하였으며, 실험군에게는 PBL-ISP 실습교육을 실시하였다.

간호대학 학생들은 개인적으로 졸업할 때갂지 총 8회의 시뮬레이션 실습교육을 받는다. 학생들은 1학기부터 8주간의 교내 이론수업을 마치고, 교내 시뮬레이션 실습 평가에서 통과하면 이론과 시뮬레이션 실습교육을 받은 전공 교과목 내용과 관련된 부서에서 8주 동안 임상 실습을 한다. 캘리포니아주에서는 시뮬레이션 실습 평가에서 통과하지 못하면 병원에서 임상 실습을 허락하지 않는다. 본 연구에서는 PBL-ISP 교육 4개의 모듈을 학기별로 적용하여 운영하였다.

2019년 가을 학기 동안 총 8회의 시뮬레이션 실습이 실습계획표대로 오전 8시에서 오후 4시까지 진행된다. 이 시간 중 오전 8시에서 9시까지는 교수자 미팅을 하고, 점심 시간 1시간을 제외하면 학생은 총 6시간 동안 실습교육을 받는다.

실험군에게 적용한 PBL-ISP 교육 프로그램은 5세션의 10단계로 운영한다. 세션 1은 prerequisite learning으로 1단계가 포함된다. 세션 2는 2, 3단계이며 소요시간은 2시간이다. 세션 3에서는 4단계가 1시간 동안 진행되며, 세션 4에서는 5–8단계가 3시간 동안 이루어진다. 마지막 세션 5에서는 9, 10단계가 진행된다.

반면에 대조군 학생 3, 4명으로 한 팀을 이루고 총 3, 4개의 팀으로 나뉘어 고충실도 시뮬레이터를 활용한 시뮬레이션 실습교육을 4세션으로 운영한다. 세션 1은 사전 테스트를 실시하고 실습 오리엔테이션(시뮬레이션 진행과정과 시나리오에 대한 설명 등)을 1시간 동안 실시한다. 세션 2에서는 시나리오에서 간호사가 중재해야 할 핵심술기에 대한 훈련을 팀별로 교수의 감독 하에 1시간 동안 진행하며, 세션 3에서는 고충실도 시뮬레이터를 활용하여 팀별 15분 이내로 시뮬레이션을 구현하여 1시간이 소요된다. 세션 4는 3시간 동안 디브리핑과 평가회를 진행한다. 이 시간 동안 시뮬레이션 실행을 하는 팀 외의 학생들은 강의실에 대기하고 시뮬레이션 구현이 끝나면 다른 디브리핑 장소로 이동하여 디브리핑 준비를 한다. 디브리핑 세션에서는 모든 학생들이 팀별로 준비한 내용을 발표하고, 각자의 경험 및 강점과 약점에 대한 자아성찰을 한다. 마무리는 교수자가 시나리오 상황에 대한 재반영을 통해 내용을 정리해주고, 간호과정에 대한 조언 등을 한다.

### 자료 수집

자료수집은 1학기에서 4학기 학생 총 82명을 대상으로 총 8개의 모듈을 운영하는 M 대학 시뮬레이션 실습실에서 진행하였다. 자료수집 기간은 2019년 가을 학기이며, 8월 20일부터 12월 3일까지 수집하였다. 자료수집 전에 간호대학 학과장과 시뮬레이션 실습 담당 교수 및 과목 담당 교수와의 회의에서 연구목적과 절차를 설명하고 동의를 얻었다. 시뮬레이션 담당 교수가 시뮬레이션 수업을 시작하기 전에 연구목적과 설문지를 설명한 후 학생들의 동의를 얻었다. 사전조사로 연령, 학기, 종교, 전공 만족도에 대해 조사하였고, 처치로 실습교육을 마친 후에 사후조사로 간호지식, 비판적 사고성향, 문제 해결 능력, 수업 몰입도 설문지를 작성하여 제출하였다.

**Figure. 2.**
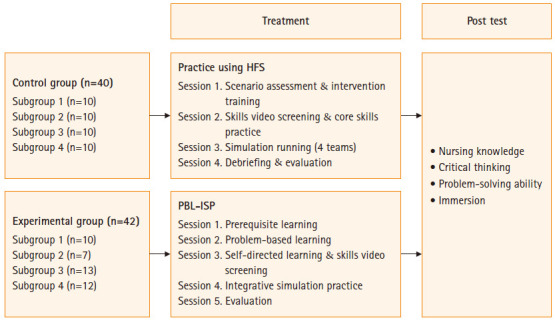
Research procedure. HFS: High-fidelity simulation; PBL-ISP: problem-based learning–integrative simulation practice.

자료수집은 시뮬레이션 실습계획표에 따라 진행하였으며, 대조군과 실험군의 실습 간격이 짧은 경우 사전조사가 실험의 확산을 일으킬 수 있다고 생각하여 동일 학기 학생 대조군에게 먼저 고충실도 시뮬레이터를 활용한 실습교육을 하고 사후조사를 하였다. 그 다음은 동일 학기 다른 학생 실험군에게 PBL-ISP 교육을 적용한 후 사후조사를 실시하였다(Figure 2).

### 자료 분석 방법

수집된 자료는 IBM SPSS for Windows, ver. 23.0 (IBM Corp., Armonk, NY, USA) 프로그램을 이용하여 대상자의 일반적인 특성은 실수와 백분율, 평균과 표준편차를 구하였으며, 두 집단 간의 동질성 검증은 Chi-square test 또는 Fisher’s exact test와 t-test로 확인하였고, 가설 검정은 independent t-test로 분석하였다. 연구 도구의 신뢰도는 Cronbach’s α를 사용하여 분석하였다.

## Results

### 집단 간 동질성 검증

연구 대상자는 총 82명으로 실험군 42명, 대조군 40명이었으며, 실험군과 대조군의 동질성 검정 결과는 Table 1과 같다. 즉, 학기는 실험군에서 3학기 학생이 31.0%로 가장 많았고, 대조군은 학기별 25.0%로 균등하였으며, 두 집단 간 유의한 차이가 없었다. 성별과 종교, 연령에서도 두 집단 간에 유의한 차이가 없었다. 평균 연령은 23.5세였는데 실험군의 평균 연령은 24.0세, 대조군은 22.9세로 나타났으며, 두 집단 간의 유의한 차이가 없었다. 간호학 전공 만족도는 두 집단 모두 높은 점수로 나타났으며, 실험군 4.14점, 대조군 3.90점으로 두 집단 간 유의한 차이가 없었다(Table 2).

### 가설 검정

#### 제1가설

‘PBL-ISP 교육을 받은 실험군은 고충실도 시뮬레이터를 활용한 실습교육을 받은 대조군보다 간호지식 점수가 높을 것이다’라는 가설을 검정한 결과, 실험군이 20점 만점에 16.31점, 대조군이 14.40점으로 두 집단 간에 통계적으로 유의한 차이가 있는 것으로 나타나 제1가설은 지지되었다(Table 3).

#### 제2가설

‘PBL-ISP 교육을 받은 실험군은 고충실도 시뮬레이터를 활용한 실습교육을 받은 대조군보다 비판적 사고성향 점수가 높을 것이다’ 라는 가설을 검정한 결과, 실험군이 3.74점, 대조군이 3.49점으로 두 집단 간에 통계적으로 유의한 차이가 있는 것으로 나타나 제2가설은 지지되었다. 비판적 사고성향의 하위 영역을 살펴 보면, 체계성을 제외한 지적/열정/호기심, 신중함, 자신감, 지적 공정성, 건전한 회의성, 객관성에서 통계적으로 유의한 차이를 나타냈다(Table 3).

#### 제3가설

‘PBL-ISP 교육을 받은 실험군은 고충실도 시뮬레이터를 활용한 실습교육을 받은 대조군보다 문제 해결 능력 점수가 높을 것이다’라는 가설을 검정한 결과, 실험군이 3.81점, 대조군이 3.52점으로 두 집단 간에 통계적으로 유의한 차이가 있는 것으로 나타나 제3가설은 지지되었다. 문제 해결 능력의 하위 영역을 살펴 보면, 문제 인식, 정보 수집 분석, 확산적 사고 의사결정, 기획력 실행, 평가 피드백에서 통계적으로 유의한 차이를 나타냈다(Table 3).

#### 제4가설

‘PBL-ISP 교육을 받은 실험군은 고충실도 시뮬레이터를 활용한 실습교육을 받은 대조군보다 수업 몰입도 점수가 높을 것이다’라는 가설을 검정한 결과, 실험군이 3.94점, 대조군이 3.52점으로 두 집단 간에 통계적으로 유의한 차이가 있는 것으로 나타나 제4가설은 지지되었다. 수업 몰입도의 하위 영역을 살펴 보면, 원격 현존감을 제외한 즐거움, 주의집중, 관여, 시간 왜곡에서 통계적으로 유의한 차이를 나타냈다(Table 3).

## Discussion

본 연구는 PBL-ISP 교육 프로그램을 개발하고 적용하여 간호지식, 비판적 사고성향과 문제 해결 능력, 수업 몰입도에 미치는 효과를 평가하고 이들의 관계를 파악하여 간호학생의 시뮬레이션 실습교육을 효과적으로 운영할 수 있는 가이드라인을 마련하고자 시도하였다.

PBL-ISP 교육을 받은 간호학생들의 간호지식 점수가 20점 만점에 16.31점인 반면, 고충실도 시뮬레이터를 활용한 실습교육을 받은 학생들의 점수는 14.40점으로 나타났다. 이 결과는 Kim과 Chun [12]의 연구에서 문제중심학습과 시뮬레이션 실습 융합교육을 실시한 후 간호지식 점수가 통계적으로 유의한 향상을 보인 결과와 유사하였다. 연구설계에서 실습 전 점수를 확인하지 못해 이 결과를 해석하기에 무리가 있으나, 간호지식 점수가 향상된 이유는 통합 시뮬레이션 실습 전 선수 학습과 문제중심학습으로 자가주도학습의 결과로 환자의 간호문제상황을 이해하고 적용하는 데 도움이 되었기 때문이라고 생각된다.

비판적 사고성향은 PBL-ISP 교육을 받은 학생들이 5점 만점에 3.74점, 고충실도 시뮬레이터를 활용한 실습교육을 받은 학생들은 3.49점으로 통계적으로 유의한 차이를 보였다. 단, 하위 영역 중 ‘체계성’에서는 차이가 없는 것으로 나타났다. Kim과 Chun [12]은 문제중심학습과 시뮬레이션 융합교육을 실시한 단일군의 전후 비판적 사고성향이 향상되었음을 보고하였고, 표준화 환자를 활용한 시뮬레이션 실습교육을 실시한 후에도 비판적 사고성향이 높게 나타난 연구가 있다[25]. 반면에 문제중심학습을 적용한 시뮬레이션 실습교육 전후 차이를 보이지 않은 연구도 있었다[15]. 한편, 시뮬레이션 실습과 연계하지 않았지만 문제중심학습 집단이 주제중심학습을 실시한 집단에 비해 비판적 사고성향이 통계적으로 유의한 차이를 보였고, 하위 영역의 체계성에서도 유의한 효과가 있었음을 보고한 연구가 있다[26]. 따라서 문제중심학습은 학습자들이 간호 상황을 중심으로 문제 해결에 필요한 지식, 기술, 태도를 훈련하면서 비판적 사고를 독려할 수 있는 학습방법으로, 문제중심학습을 시뮬레이션 실습교육에 통합하면 비판적 사고능력을 함양시킬 수 있는 것으로 생각된다.

문제 해결 능력은 PBL-ISP 교육을 받은 학생들이 5점 만점에 3.81점, 고충실도 시뮬레이터를 활용한 실습교육을 받은 학생들은 3.52점으로 통계적으로 유의한 차이를 보였다. 이는 문제중심학습의 효과를 검증한 Kim과 Chun [12]의 연구와 유사한 결과를 나타냈으며, PBL 연계 시뮬레이션 교육 후 문제 해결 적극성이 증가한 연구결과도 있다[13,27]. 또한 표준화 환자 활용 시뮬레이션 교육이 고충실도 환자 모형 시뮬레이터를 활용한 시뮬레이션 교육보다 문제 해결 능력의 향상에 더 나은 효과가 있다고 하였다[28]. 이러한 결과가 중요한 이유는 임상에서 대상자의 상황을 이해하고, 문제를 빠르게 파악하여 해결할 수 있는 능력을 요구하기 때문이라고 생각된다. Lee와 Kim [17]의 시뮬레이션 실습교육의 효과 연구에서는 분만 시뮬레이터를 활용하여 실제 산과 간호 영역의 임상 상황과 유사한 실습 환경에서 시뮬레이션 실습 전에 비해 실습 후 지식, 정확한 사정, 적절한 중재와 적절한 의사소통, 우선순위 설정의 임상술기 수행에 대한 자신감이 유의하게 상승되었음을 보고하였다. 한편, 임상술기 수행에 대한 자신감의 하위 영역 중 ‘우선순위 설정’에서 가장 낮은 것으로 보고하였다[17]. 즉, 시뮬레이션 실습교육은 학생들에게 반복 실습의 기회를 통해 자신감과 같은 긍정적인 영향을 주는 것은 분명한 사실이다. 더욱이 학생들이 실제 상황에 있다고 인지하여 환자의 간호 문제를 파악하고, 대상자의 우선순위별로 문제를 해결하는 데 도움을 줄 수 있는 교수 전략을 병행한다면 긍정적 효과를 더 강화할 수 있다고 보여진다. 따라서 효과적인 시뮬레이션 실습교육을 운영하기 위해서는 우선 실제 임상 근거 기반의 시나리오를 신중히 고려하여 모듈을 개발하고, 다음은 시뮬레이션 실습교육 운영 계획과 교수 전략을 세우는 것이 중요할 것이다. 이에 본 연구는 시뮬레이션 실습을 담당하는 교수들이 더 이상 시행착오를 겪지 않고 학생들에게 효율적인 교수법과 개발된 모듈을 공유하는 데 기초자료를 제공한 것에 그 의의가 있다고 생각된다.

수업 몰입도는 PBL-ISP 교육을 받은 학생들이 5점 만점에 3.94점, 고충실도 시뮬레이터를 활용한 실습교육을 받은 학생들은 3.52점으로 통계적으로 유의한 차이를 보였다. 단, 하위 영역 중 ‘원격 현존감’에서는 차이가 없는 것으로 나타났다. 이는 자기조절 학습능력이 학습 몰입도에 통계적으로 유의하게 높게 나타났다고 보고한 연구[29]와 시뮬레이션 실습교육이 학습 몰입 향상에 영향을 주었다는 연구[5]에 의해 설명될 수 있다. 그러나 하위 영역의 원격 현존감은 사이버 강의 몰입 척도로 개발된 도구[24]로, 본 연구에서는 영향을 미치지 않은 것으로 나타났다고 생각된다.

이러한 결과는 문제중심학습을 통해 학생들이 스스로 지식을 습득하고 문제를 해결해가는 자기주도 학습에 의한 효과로 나타난 결과로 보여진다. 즉, PBL-ISP 교육 환경에서 문제 해결 과정을 통해 자기주도 학습능력이 향상되어 나타나는 결과로, 결국 학습자가 능동적으로 학습을 주도하고 통제하는 능력을 기르면서 학습에 몰입할 수 있다고 생각된다.

본 연구의 차별성을 살펴보면, 첫째, PBL-ISP 교육을 6시간 동안 5세션의 10단계 프로그램으로 세팅하였다. 둘째, PBL 모듈과 ISP 모듈을 개발하여 통합 시뮬레이션 실습교육을 하였다. 셋째, 디브리핑 단계에서 시뮬레이션 실행 직후 표준화 환자와 평가자 1인과 한 개의 팀이 실시하는 간소 디브리핑과 표준화 환자를 제외한 모든 학생과 교수자들이 참여하여 실시하는 통합 디브리핑을 실시하였다. 넷째, 평가를 실시한 교수자들이 학생 평가 결과를 공유하였다. 다섯째, 시뮬레이션 실습 운영에 필요한 인력 풀이 구축되어 있었다. 즉, 전공과목 담당 교수 4인, 시뮬레이션 전임 교수 1인, 시뮬레이션 실습실 직원 2명, 학기별 instructor 4인, 임상 간호사 5인, 표준화 환자 봉사자 10명이 있었다. 기타 PBL-ISP 교육 단계 외에 우리나라와의 다른 점은 대학의 시뮬레이션 실습과 임상 실습의 연계성으로, 본 연구에서는 대학의 시뮬레이션 실습 평가에 패스한 학생들이 임상 실습을 할 수 있는 시스템을 갖추고 있었다. 이러한 시스템은 결국 학생들이 시뮬레이션 실습에 참여하기 위해 철저히 준비하도록 동기화하였고, 이러한 자세와 태도는 간호사로서 갖추어야 할 역량을 기르는 데 긍정적인 효과를 주었다. 이것은 우리나라의 실정에 도입하기에는 무리가 있다고 보여진다. 그러나 추후 이러한 시스템을 도입하면 시뮬레이션 실습의 체계화를 위한 모듈 개발, 환경 구축, 대학과 임상의 협력, 시뮬레이션 교수법 등의 내실화에 기여할 것이며, 시뮬레이션 실습 시간을 임상 실습 시간으로 일부 인정받는 데 기여할 수 있을 것이다. 따라서 본 연구에서 제시한 PBL-ISP 교육 프로그램은 체계적인 시뮬레이션 실습 시스템을 구축하는 데 기초를 제공하였다고 생각된다.

끝으로 PBL-ISP 교육 프로그램은 부서와 병동 및 환자의 증상과 상황에 핵심 간호술기를 접목하여 모듈을 개발하고 실습교육으로 적용하였다는 점과, 임상 간호사를 포함한 다양한 평가자가 학생에 대한 논의를 하는 평가 공유를 통해 교수자의 객관적 평가 역량을 기를 수 있다는 것에 본 연구의 의의가 있다고 생각한다. 한편, 이러한 연구의 의의가 있음에도 몇 가지 제한점을 고려해 보고자 한다. 본 연구는 비동등성 대조군 사후시차 설계이므로 실험 전과 비교하지 못해 연구결과의 해석에 제한이 있다. 또한 시뮬레이션 실습에 참여하는 학생 대 평가자 비율이 2:1 정도로 운영되었다. 따라서 평가자 간의 타당도와 신뢰도를 검증하여 주관성을 배제할 필요가 있다고 생각한다. 추후 연구에서는 이러한 제한점을 고려하여 PBL-ISP 교육의 효과를 분석하면 PBL-ISP 교육 프로그램을 널리 활용하는 데 도움을 줄 수 있을 것이다.

## Conclusion

본 연구는 간호학생들에게 PBL-ISP 교육을 적용하여 간호지식, 비판적 사고성향, 문제 해결 능력 및 수업 몰입도에 미치는 효과를 파악하고자 시도하였다. 최근 간호대학에서 임상 실습을 보완하기 위해 시뮬레이션 실습교육의 확대 방안을 고려하고 있다. 특히 관찰 위주의 임상 실습에서 제한된 부분을 극복하기 위해 시뮬레이션을 통한 실습교육 프로그램의 확대 적용이 필요한 시기이다. 이에 본 연구를 통해 PBL-ISP 교육이 간호학생들의 간호지식, 비판적 사고성향, 문제 해결 능력 및 수업 몰입도에 효과가 있음을 파악하였다. 실험처치 후 본 연구대상의 학생들에게 공평한 수업을 제공하기 위해 추후 해야 할 실습교육에서 PBL-ISP교육 프로그램을 적용하기로 교수자들에게 연구결과 및 모듈을 공유하고 협조를 구하였다. 본 연구는 한 간호대학의 학생들을 대상으로 한 결과이므로 일반화시키기에는 무리가 있으나, PBL-ISP 교육이 효과적인 교육방법임을 확인하고 활용하는 기초 자료가 될 수 있을 것이다.

## Figures and Tables

**Table 1.** Content of PBL-ISP modules and nursing skills

Semester	Module	Core nursing skills	HFS/SP
First	A 68-year-old woman diagnosed 40 years ago with HTN has an appointment to see her primary care provider (in the ambulatory clinic)	Obtaining a history and performing an assessment	SimMan & SP
		General physical assessment	
		Vital signs	
		Administering oral and topical medications	
Second	OB post-epidural nursing care with fetal deceleration, delivery care, and newborn care (in the delivery room and nursery room)	Inserting a Foley catheter	SimMom & SimBaby
		Administering oxygen	
		Immunizations for children from birth through 6 years old	
		Subcutaneous injection	
		Postpartum and newborn assessment	
Third	Ischemic stroke with type 2 DM and HTN (in the intensive care unit)	EKG, CPR, and transfusion	SimMan & SP
		Glucose point-of-care testing	
		Administering intravenous push	
		Administering intravenous piggyback medications	
Fourth	Alzheimer’s disease, multiple sclerosis (in the neurology clinic)	Well elder assessment	SP with dementia equipment
		Personal hygiene (transfer, positioning, ambulation)	
		Nutrition	
		Using the SBAR method when communicating with hea care professionals	

CPR: Cardiopulmonary resuscitation; DM: diabetes mellitus; EKG: electrocardiogram; HFS: high-fidelity simulator (SimMom, SimBaby, SimMan; Laerdal Medical, Stavanger, Norway); HTN: hypertension; PBL-ISP: problem-based learning - integrative simulation practice; SBAR: situation, background, assessment, recommendation; SP: standardized patient (a real person).

**Table 2.** Homogeneity of characteristics between the experimental and control groups (N=82)

Variable	Categories	n (%) or Mean ± SD		χ²/t	*p*
		Experimental (n = 42)	Control (n = 40)		
Semester	First	10 (23.8)	10 (25.0)	1.05	.795
	Second	7 (16.7)	10 (25.0)		
	Third	13 (31.0)	10 (25.0)		
	Fourth	12 (28.6)	10 (25.0)		
Gender	Man	8 (19.0)	12 (30.0)	1.33^†^	.308
	Woman	34 (81.0)	28 (70.0)		
Religion	None	21 (50.0)	21 (52.5)	3.69^†^	.321
	Protestantism	16 (38.1)	11 (27.5)		
	Catholicism	1 (2.4)	5 (12.5)		
	Buddhism	4 (9.5)	3 (7.5)		
Age (year)	19–21	23 (54.8)	17 (42.5)	2.71^†^	.458
	22–24	11 (26.2)	14 (35.0)		
	25–29	3 (7.1)	6 (15.0)		
	30–47	5 (11.9)	3 (7.5)		
		24.0 ± 6.44	22.9 ± 2.72	1.11	.272
Satisfaction with nursing		4.14 ± 0.72	3.90 ± 0.74	1.50	.137

† Fisher exact test.

**Table 3.** Comparisons of nursing knowledge, critical thinking, problem-solving ability, and immersion scores between the experimental and control groups after the intervention (N=82)

Variable	Categories	Mean±SD		t	*p*
		Experimental (n=42)	Control (n=40)		
Nursing knowledge		16.31 ± 2.11	14.40 ± 2.57	3.67	< .001
Critical thinking	Intellectualism/passion/curiosity	3.93 ± 0.34	3.69 ± 0.29	3.40	.001
	Deliberation	3.87 ± 0.48	3.57 ± 0.48	2.94	.004
	Confidence	3.72 ± 0.46	3.35 ± 0.51	3.45	.001
	Systematic	3.13 ± 0.52	3.34 ± 0.54	–1.76	.082
	Intellectual fairness	3.76 ± 0.45	3.38 ± 0.52	3.51	.001
	Sound skepticism	3.94 ± 0.49	3.65 ± 0.40	2.93	.004
	Objectivity	3.81 ± 0.61	3.54 ± 0.49	2.19	.032
	Total	3.74 ± 0.31	3.49 ± 0.36	3.40	.001
Problem-solving ability	Problem recognition	3.98 ± 0.42	3.52 ± 0.35	3.65	< .001
	Information collection analysis	3.90 ± 0.38	3.56 ± 0.44	3.74	< .001
	Diffuse incident decision-making	3.70 ± 0.43	3.42 ± 0.47	2.82	.006
	Planning power execution	3.67 ± 0.51	3.39 ± 0.49	2.57	.012
	Feedback	3.82 ± 0.43	3.60 ± 0.44	2.27	.026
	Total	3.81 ± 0.36	3.52 ± 0.36	3.52	.001
Immersion	Joy	4.49 ± 0.53	3.88 ± 0.38	5.97	< .001
	Remote presence	4.10 ± 0.59	3.86 ± 0.66	1.77	.081
	Concentration	4.17 ± 0.59	3.74 ± 0.49	3.58	.001
	Engagement	3.39 ± 0.75	3.06 ± 0.64	2.08	.040
	Time distortion	3.45 ± 0.74	3.08 ± 0.64	2.47	.016
	Total	3.94 ± 0.43	3.52 ± 0.42	4.44	< .001

## References

[1] Thomas RE (1997). Problem-based learning: measurable outcomes. Med Educ.

[2] Cannon C, Schell K, Duch B, Groh S, Allen D (2001). The power of problem-based learning.

[3] Kang IA (2003). PBL theory and practice.

[4] Ma RW, Lee EJ, Kim HO, Jee YJ (2017). Study on the simulation-based education using the self-directed learning. Asia-pacific J Multimed Serv Converg Art Humanit Sociol.

[5] Kim MO, Lee AY, Nam HA (2017). Effects on nursing students’ learning flow, critical Judgement, and problem-solving ability in simulation training: focused on obstetrical nursing case. J Learner-Cent Curric Instr.

[6] Eom MR, Kim HS, Kim EK, Seong KY (2010). Effects of teaching method using standardized patients on nursing competence in subcutaneous injection, self-directed learning readiness, and problem-solving ability. J Korean Acad Nurs.

[7] Yoo MS, Yoo IY, Park YO, Son YJ (2002). Comparison of student’s clinical competency in different instructional methods for fundamentals of nursing practicum. J Korean Acad Nurs.

[8] Kneebone R (2005). Evaluating clinical simulations for learning procedural skills: a theory-based approach. Acad Med.

[9] Ackermann AD, Kenny G, Walker C (2007). Simulator programs for new nurses’ orientation: a retention strategy. J Nurses Staff Dev.

[10] Turcato N, Roberson C, Covert K (2008). Simulation-based education: what’s in it for nurse anesthesia educators?. AANA J.

[11] Song YA (2014). Effect of simulation-based practice by applying problem based learning on problem solving process, self-confidence in clinical performance and nursing competence. Korean J Women Health Nurs.

[12] Kim HJ, Chun IH (2018). The effect of problem-based learning and simulation practice convergence education for nursing students. J Korea Converg Soc.

[13] Park MJ, Choi D (2018). The effect of simulation integrated with problem based learning on system thinking, learning flow, proactivity in problem solving and performance ability for medication in nursing students. J Digit Converg.

[14] Cho OH, Hwang KH (2017). The effect of education based on simulation with problem-based learning on nursing students’ learning motivation, learning strategy, and academic achievement. J Korea Contents Assoc.

[15] Kim JS, Kim YH (2016). The effects of simulation practice education applying problem-based learning on problem solving ability, critical thinking and learning satisfaction of nursing students. J Korea Contents Assoc.

[16] Kim JI, Kang H, Park S, Ahn S (2014). Current status of women’s health nursing practicum and direction. Korean J Women Health Nurs.

[17] Lee WS, Kim M (2011). Effects and adequacy of high-fidelity simulation-based training for obstetrical nursing. J Korean Acad Nurs.

[18] Ellis D, Crofts JF, Hunt LP, Read M, Fox R, James M (2008). Hospital, simulation center, and teamwork training for eclampsia management: a randomized controlled trial. Obstet Gynecol.

[19] Ryoo EN, Ha EH, Cho JY (2013). Comparison of learning effects using high-fidelity and multi-mode simulation: an application of emergency care for a patient with cardiac arrest. J Korean Acad Nurs.

[20] Gates MG, Parr MB, Hughen JE (2012). Enhancing nursing knowledge using high-fidelity simulation. J Nurs Educ.

[21] Crea KA (2011). Practice skill development through the use of human patient simulation. Am J Pharm Educ.

[22] Yoon J (2004). Development of an instrument for the measurement of critical thinking disposition: in nursing [dissertation].

[23] Lee SJ, Jang YG, Lee HN, Park GY (2003 Dec). A study on the development of life-skills: communication, problem solving, and self-directed learning.

[24] Shin NM, Kim KS, Kim KY (2005). An empirical study on the cyber-class flow model. Korean J Educ Res.

[25] Hwang EH, Kim KH (2016). Differences in critical thinking disposition and life-skills of nursing students: comparison of high school tracks. J Korean Soc Wellness.

[26] Song YA (2008). Comparison of learning satisfaction, critical thinking disposition, learning attitude and motivation between PBL and SBL groups. J Korean Acad Soc Nurs Educ.

[27] Kim SH, Park IS (2015). Effects of simulation practice by applying problem based learning on the critical thinking disposition, problem-solving process and self-confidence of nursing process in nursing students. J Korea Soc Simul Nurs.

[28] Choi E, Kwag Y (2015). Nursing students’ problem solving, and clinical competence between standard patient and high fidelity simulator simulation. Asia-pacific J Multimed Serv Converg Art Humanit Sociol.

[29] Joo YJ, Kim JY, Choi HR (2009). Investigating the structural relationship among self-regulated learning, learning flow, satisfaction and learning persistence in corporate e-Learning. J Educ Technol.

